# The comparison of pegylated liposomal doxorubicin and beta-carotene effects on JAR and JEG-3 choriocarcinoma human cell culture models

**DOI:** 10.4274/jtgga.galenos.2020.2019.0199

**Published:** 2020-09-03

**Authors:** Seyit Ahmet Erol, Görker Sel, Mehmet İbrahim Harma, Müge Harma, İshak Özel Tekin

**Affiliations:** 1 Clinic of Obstetrics and Gynecology, Ankara City Hospital, Ankara, Turkey; 2 Clinic of Obstetrics and Gynecology, Zonguldak Bülent Ecevit University Health Practice and Research Hospital, Zonguldak, Turkey; 3 Clinic of Obstetrics and Gynecology, Division of Gynecologic Oncology, Zonguldak Bülent Ecevit University Health Practice and Research Hospital, Zonguldak, Turkey; 4 Clinic of Immunology, Zonguldak Bülent Ecevit University Health Practice and Research Hospital, Zonguldak, Turkey

**Keywords:** Pegylated liposomal doxorubicin, beta-carotene, choriocarcinoma, JAR cell culture, JEG-3 cell culture

## Abstract

**Objective:**

The aim was to investigate the effectiveness of pegylated liposomal doxorubicin (PLD), beta-carotene, and a combination of PLD and beta-carotene on JAR and JEG-3 human choriocarcinoma (CC) cell lines for the treatment of CC.

**Material and Methods:**

JAR and JEG-3 cells were cultured. PLD and beta-carotene trial groups were determined with different doses (for single drug trial; PLD 1, 2, 5 μg/mL and beta-carotene 1, 5, 10 μg/mL, and for combined drug trial; all PLD doses combined with beta-carotene 5 μg/mL). Drugs were administered to cultures simultaneously, and 72 hours later the cells were detached using trypsin-ethylenediamine tetraacetic acid solution. The percentage of apoptotic cells was determined by flow cytometry after annexin V staining. One set of the supernatant was collected before trypsin application to investigate beta-human chorionic gonadotropin (β-hCG) and hyperglycosylated hCG (H-hCG) levels. Statistical analyses of the apoptotic ratios were performed using Shapiro-Wilk, Kruskal-Wallis and Mann-Whitney U tests.

**Results:**

Apoptosis increased in JAR and JEG-3 cultures after treatment with all doses of PLD (p<0.05). A single application of each beta-carotene dose increased apoptosis in JAR cells (p<0.05) but had no apoptotic effects on JEG-3 cells. In the PLD and beta-carotene combination group, apoptosis increased in both JAR and JEG-3 cells (p<0.05).

**Conclusion:**

To our knowledge, this is the first investigation of the effectiveness of PLD, beta-carotene, and PLD + beta-carotene combination therapy in two different CC cell lines. PLD is a promising chemotherapeutic drug, and beta-carotene can be used as a novel non-chemotherapeutic agent for treatment of CC. Based on the results of this study, vitamin A supplementation may have promise as a preventive measure. However, these data need support from animal experiments and clinical trials.

## Introduction

Gestational trophoblastic disease (GTD) appears in the fetal chorion and involves a variety of interrelated diseases. The effects of GTD range from benign hydatidiform moles (HM), which usually resolve spontaneously, to life-threatening. GTD usually develops from HM, but it has been observed in aborted, term, and ectopic pregnancies. Choriocarcinoma (CC) is the most aggressive histologic type of GTD (1). The most common clinical biomarker for the diagnosis and follow-up of this disease is serum beta-human chorionic gonadotropin (β-hCG). β-hCG follow-up should be carried out in series in terms of the course and behavior of the disease and after the treatment period. However, hCG produced in CC has a larger oligosaccharide side chain than hCG synthesized in normal pregnancy and is known as hyperglycosylated hCG (H-hCG) (2).

There are many medical treatments for CC depending on the stage of the disease. Multi-agent chemotherapy protocol is the preferred treatment method if hCG levels increase during treatment, if metastasis develops, or a resistance develops to sequential single-agent chemotherapy protocol (3). The current evidence-based initial therapy in treatment of high-risk metastatic GTD is the etoposide, methotrexate, actinomycin D, cyclophosphamide, vincristine (EMA-CO) protocol. Drug resistance may develop during or after primary chemotherapy in approximately 20% of high risk gestational trophoblostic neoplasia patients. In patients who do not respond to first-line EMA-CO therapy or for those who relapse, the most appropriate second-line therapy is the etoposide, methotrexate, actinomycin D, etoposide, cisplatin (EMA-EP) protocol. However, this protocol is quite toxic (4). The International Society of the Study of Trophoblastic Diseases has reported that different treatment regimens such as paclitaxel/etoposide may be effective in relapsing patients after paclitaxel/cisplatin based combination therapies, but more studies should be conducted in this regard (5).

Doxorubicin is an anthracycline antibiotic which intercalates between base pairs in the DNA helix, thereby preventing DNA replication and ultimately inhibiting protein synthesis (6). Pegylated liposomal doxorubicin (PLD) is a polyethylene glycol-coated form of doxorubicin that has fewer side effects than those of doxorubicin, and it is approved for the treatment of HIV-related Kaposi’s sarcoma, metastatic breast cancer, advanced ovarian cancer and multiple myeloma (7).

Beta-carotene is a naturally-occurring retinol (vitamin A) precursor obtained from certain fruits and vegetables with potential antineoplastic and chemopreventive activities. It is particularly protective in chemical carcinogenesis by taking part in the detoxification of peroxide radicals (8). It was reported that the incidence of complete hydatidiform mole decreased due to increase in carotene consumption. Parazinni et al. (9) suggested that low beta-carotene consumption is related to GTD.

The options that can be used against multiple drug resistance in the treatment of CC are limited in the literature. Therefore, the aim of this study was to investigate the effects of PLD and beta-carotene and treatment efficacy on cell culture CC models using JAR and JEG-3 cell cultures in order to provide more effective treatment methods by providing a new perspective in CC treatment.

## Material and Methods

### JAR and JEG-3 cell culture lines

This study was planned as pre- and post-test study. JAR and JEG-3 cell culture lines were obtained from the American Tissue Type Culture Collection. All cell cultures were maintained and cultured in Roswell Park Memorial Institute (RPMI) - 1640 medium (INTERLAB Laboratory Products, İstanbul, Turkey) supplemented with 10% heat-inactivated fetal calf serum, (DATEKS Technical Systems, Ankara, Turkey) penicillin streptomycin, and L-glutamine (BRK Chemistry and Biotechnology, İzmir, Turkey) in a 98% humidified, 5% CO2 atmosphere at 37 °C in a Nuve EC 160 CO_2_ incubator in 75 cm^2^ flasks.

### Pegylated liposomal doxorubicin preparation

PLD was purchased from Sigma-Aldrich Chemie GmbH, Germany, prepared in dosages of 1, 2 and 5 μg/mL by diluting in dimethyl sulfoxide (DMSO) (DATEKS Technical Systems, Ankara, Turkey), and diluted in RPMI-1640 to a maximum concentration such that DMSO formed less than 1% of the mixture.

### Beta-carotene preparation

Beta-carotene was purchased from Sigma-Aldrich Chemie GmbH, Germany, prepared in dosages of 1, 5 and 10 μg/mL by diluting in DMSO, and diluted by RPMI-1640 to a maximum concentration such that DMSO formed less than 1% of the mixture.

### Preparation of chemotherapeutics for tests, β-hCG and H-hCG measurement

The dosages used in the PLD and beta-carotene trial groups were as follows:

Single drug trial: PLD 1, 2 and 5 μg/mL; beta-carotene 1, 5 and 10 μg/mL.

Combined PLD and beta-carotene drug trial: PLD 1, 2 and 5 μg/mL; and beta-carotene 5 μg/mL.

Drugs were administered to the cells simultaneously, and 72 hours after drug administration, the cells were detached from the bottom of plate by using trypsin ethylenediamine tetraacetic acid (EDTA) solution. The degree of apoptosis was determined by flow cytometry (FCM). The supernatant was collected before trypsin application from one set of samples from each experiment and stored at -80 °C frozen to investigate H-hCG levels. H-hCG levels were investigated using an immunoenzymatic method (Sunred Elisa Kit and DXI 600, Beckman Coulter, CA, USA). All tests were repeated six times.

### Detection of apoptosis using annexin V

The annexin V-binding assay is one of the most sensitive and widely used techniques to detect and distinguish between early apoptosis and late apoptosis, as well as between apoptosis and necrosis. Annexin V is a protein that binds preferentially to phosphatidylserine, which is located at the outer surface of the cell membrane. This feature allows apoptotic cells to be observed after marking them with a fluorescent agent such as fluorescein isothiocyanate (FITC) (10). The binding ratio of FITC-annexin-V complex to phosphatidylserine at the cell membrane can be measured using FCM.

Ethics committee approval was received for this study from the Ethics Committee of Bülent Ecevit University Faculty of Medicine (approval number: 2014-68-25/03, date: 03/25/2014). No informed consent was obtained due to cell culture study.

### Statistical analysis

Statistical analyses of degree of apoptosis were performed with the SPSS, version 19.0 package program. Descriptive statistics related to continuous variables are given as median and range (minimum and maximum values). The conformity of variances to normal distribution was evaluated through the Shapiro-Wilk test. The Kruskal-Wallis test was used in quaternary comparison of doses and the Mann-Whitney U test and the Mann-Whitney U test with Bonferonni correction were used in binary subgroup comparison between doses. In all statistical analysis in the study, comparisons below a p-value of 0.05 were accepted as statistically significant.

## Results

The median apoptotic ratio in the control group, in which only DMSO was used, was 0.3% in the JAR cell culture and was 3.7% in the JEG-3 cell culture. The median apoptotic ratios after the application of 1, 2 and 5 μg/mL PLD were; 1.3, 1.4 and 2% in the JAR cells, respectively. The increase in the apoptotic ratio was statistically significant (p<0.05) (Table 1, Graph 1). The median apoptotic ratios after the application of 1, 2 and 5 μg/mL PLD were; 16.1, 31.4 and 27.5% in the JEG-3 cells, respectively. The increase in the apoptotic ratio was also statistically significant (p<0.05) (Table 1, Graph 1).

After 72 hours, median β-hCG levels in JAR and JEG-3 cell cultures were respectively, 126 and 118 mlU/mL. Median H-hCG levels in JAR and JEG-3 cell cultures were respectively, 63 and 66 mlU/mL (Table 2). After the application of 1, 2 and 5 μg/mL PLD to JAR cell lines, median β-hCG levels were: 115, 106 and 117 mlU/mL, respectively; median H-hCG levels were: 64, 60 and 64 mlU/mL, respectively (Table 2); in JEG-3 cell lines, median β-hCG levels were: 127, 114 and 118 mlU/mL, respectively and median H-hCG levels were: 63, 61 and 59 mlU/mL, respectively (Table 2). Median β-hCG and H-hCG levels after application of PLD were found to be statistically similar despite increasing PLD dose (p>0.05).

The median apoptotic ratios after the application of 1, 5 and 10 μg/mL beta-carotene were 0.35, 0.6, and 0.8% in the JAR cells, respectively. The increase in the apoptotic ratio was statistically significant (p<0.05) (Table 3, Graph 2). The median apoptotic ratios after the application of 1, 5 and 10 μg/mL beta-carotene were 3.7, 2.5, and 2.9% in the JEG-3 cells, respectively. The increase in the apoptotic ratio was found to be statistically similar despite increasing beta-carotene dose (p>0.05) (Table 3, Graph 2).

After the application of 1, 5 and 10 μg/mL beta-carotene to JAR cell lines, median β-hCG levels were: 107, 115 and 111 mlU/mL, respectively; median H-hCG levels were: 64, 68, and 56 mlU/mL, respectively (Table 2). In JEG-3 cell lines, median β-hCG levels were: 116, 121 and 115 mlU/mL, respectively and median H-hCG levels were: 63, 63, and 64 mlU/mL, respectively (Table 2). Median β-hCG and H-hCG levels after application of beta-carotene were found to be statistically similar despite increasing beta-carotene dose (p>0.05).

The median ratios of apoptosis were 1.3, 1.4 and 2% after application of 1, 2 and 5 μg/mL PLD in JAR cell cultures, respectively; as mentioned above. With the addition of 5 μg/mL beta-carotene to those PLD doses, and after application of 5 μg/mL beta-carotene combined with 1, 2 and 5 μg/mL PLD, the median apoptotic ratios were 0.4%, 1% and 1%, respectively. This incremental increase of the combined doses were statistically significant in comparision with the control group (p<0.05), but the incremental increase in doses of PLD alone were statistically significant compared to 5 μg/mL beta-carotene combined with PLD (p<0.05) (Table 4, Graph 3). However combination of PLD and beta-carotene had no effect on β-hCG and H-hCG levels in JAR cell cultures which were between 54-63 IU/mL for H-hCG and 107-111 IU/mL for β-hCG (p>0.05) (Table 2).

The median ratios of apoptosis were 16.1, 31.4 and 27.5% after application of 1, 2, and 5 μg/mL PLD in JEG-3 cell cultures, respectively; as mentioned above. With the addition of 5 μg/mL beta-carotene to those PLD doses, and after application of 5 μg/mL beta-carotene combined with 1, 2, and 5 μg/mL PLD, the median apoptotic ratios were 6.1%, 5.7% and 15.5%, respectively. This incremental increase of the combined doses were statistically significant in comparision with the control group (p<0.05), but the incremental increase when PLD was used alone were statistically significant in comparision to the combination of PLD at different concentrations and 5 μg/mL beta-carotene (p<0.05) (Table 4, Graph 3). After treatment with PLD and beta-carotene in JEG-3 cell cultures, H-hCG and β-hCG levels were between 52-63 IU/mL and 109-117 IU/mL, respectively (p<0.05) (Table 2).

## Discussion

Cell culture studies are frequently employed, especially in the evaluation of newer drugs. This is because it enables determining the effects caused by candidate drugs on molecular targets, which are common or are exptected to be effected by those drugs, and predicting the effects that the drug will have on target tissue. Recurrent tumor growth due to unpredicted changes caused by newly developed cancer drug components on cell phenotype may lead to unexpected results in clinical practice. The use of human monolayer cancer cell lines is fairly common and an important method for understanding the causes of these unexpected results obtained in clinical practice (11). JAR and JEG-3 cells are frequently used in CC cell culture studies, so these cells lines were chosen for our study.

Various drugs have cytotoxic effects on cells through either necrosis or apoptosis or a combination of these. It is a preferred characteristic of drugs used or being developed to be used in cancer treatment that the cytotoxic effects function in apoptotic pathways, as one of the mechanism leading to cancer is disruption of the normal apoptotic mechanism of a pre-cancerous cell, and domination of anti-apoptotic signal pathways of the cell on apoptotic pathways. Thus, an aim of this study was to analyze the apoptotic effect due to the use of PLD and beta-carotene, alone or in combination. In this study, the degree of apoptosis of JAR and JEG-3 cells was identified with FCM.

GTD is an interrelated group of tumors, characterized by abnormal proliferation of placental trophoblasts (12). Benign GTD consists of placental site nodules, exaggerated placental site and hydatidiform mole (complete or partial). There is potential for local invasion and distant metastasis. Malign GTDs are a persistent form of GTD usually CC, which may be grouped into three types: gestational trophoblastic neoplasia (GTN); placental site trophoblastic tumor (PSTT); and epithelioid trophoblastic tumor, which is a variation of PSTT (13).

Complete molar pregnancy risk increases in those with dietary deficiency of vitamin A (carotene) and animal fat, while there is no increase in partial molar pregnancy incidence (9). CC is seen once in every 50.000 pregnancies (14). Advance maternal age, previous molar pregnancy and blood type A carrier increase the risk (15). Patients with stage 4 or stage 2-3 and risk score of >7 are accepted as high risk and they are aggressively treated with multiple agent chemotherapy and/or adjuvant radiotherapy/surgery. Remission and survival rates in high-risk patients have improved with use of the EMA-CO protocol (16). EMA-CO protocol is the first-line treatment, based on recent evidence in metastatic GTD in high-risk patients. The optimum second-line treatment in patients not responding to first-line treatment, defined as a low plateau in measured hCG or relapsing levels of hCG after complete response, is the EMA-EP protocol. Cyclophosphamide and vincristine present in the EMA-CO protocol are replaced by etoposide and cisplatin in EMA-EP protocol (4). At present, the clinical use of PLD in CC appears to be limited and only in selected cases.

All forms of GTD are characterized by β-hCG increase due to the interrelated, heterogeneous structure of GTD, especially arising from trophoblastic epithelium of the placenta, and gestational tissue is responsible for pathogenesis of GTD. HCG is used as a biomarker for diagnosis and follow-up of the disease and response to treatment. H-hCG is a glycosylated variant of β-hCG with a larger side chain produced by invasive cytotrophoblast cells in pregnancy implantation, GTD and CC. An increase in H-hCG levels has been reported in testicular and ovarian germ cell tumors (17). Thus, the effect of beta-carotene and PLD on CC cell culture lines were measured by using both β-hCG and H-hCG together with the ratio of measures of apoptosis. This is the first report of the effects of the combination of beta-carotene and PLD on JAR and JEG-3 and the measurement of β-hCG and H-hCG by immunoenzymatic methods.

Cole et al. (17) showed in their study analyzing the biological function of H-hCG in BeWo, JAR, and JEG-3 cell culture and isolated gravid cytotrophoblast cells that H-hCG values were 841 ng/mL, 126 ng/mL, 165 ng/mL and 2.3 ng/mL, respectively and thus H-hCG was a biological tumor marker indicating cell invasion in active CC. Rubin et al. (18) evaluated the usability of H-hCG in the differentiation of benign parathyroid disease (n=18) and parathyroid cancer (n=8) and reported that H-hCG levels in the patient group with cancer were higher than 3.77 pmol/L which was the maximum concentration for patients with benign primary hyperparathyroid and thus, H-hCG could be a possible tumor marker.

PLD is a long-release formulation consisting of doxorubicin hydrochloride contained in pegylated liposomes. In breast cancer patients with increased cardiac risk PLD is administered and is also used in advanced ovarian cancer cases when platinum-based chemotherapy is unsuccessful and acquired immuno-deficiency syndrome with Kaposi sarcoma. Embedding doxorubicin into liposomes causes changes in pharmacokinetics and biodistribution and is reported to decrease toxic effects (19). Soininen et al. (20) investigated quantitative cellular intake and toxicities of doxorubicin and other liposomal forms with different concentrations (0.5 and 5 μM) in human placental CC cell culture (BeWo). These authors reported that fetal exposure decreased due to the pegylated formulation with lower cellular intake and toxicity of PLD compared to doxorubicin.

Eetezadi et al. (21) evaluated the intratumoral penetration and efficacy of block copolymer micella doxorubicin (BMC-DOX) formulation, with similar toxicity to PLD measured by the effects on microscopic lesions in HEYA8, OV-90 and SKOV3 human ovarian cell cultures, for second-line treatment of recurring ovarian cancer after cytoreduction that BCM-DOX in increasing doses between 2 mg/mL and 7.6 mg/mL provided nine-fold greater monolayer cytotoxicity compared to PLD. These authors suggested in vivo studies were required to confirm their in vitro results.

Hanf et al. (22) evaluated oxidative stress, apoptosis, phosphoproteome and epigenome changes due to doxorubicin in a cardiomyocyte cell culture model of doxorubicin cardiotoxicity. They reported that caspase-3 and fractin, which are markers of apoptosis, and 3-nitrotyrosine and malondiadehyde, which are markers of oxidative stress, increased in a dose-dependent manner following administration of (H9c2) doxorubisin at 24 and 48 hours at concentrations of 1 and 5 μM in cultured rat myoblasts and in addition histone-3 acetylation decreased. They concluded that apoptosis related to oxidative stress caused cell death in this model.

Popadiuk and Power (23) showed that there was a complete response, especially against brain metastasis, when PLD only was administered in 2-3 cycles in two patients with multiple organ metastatis recurring after standard chemotherapy and radiotherapy regimens and concluded that PLD was an active agent in high-risk CC. Essel et al. (24) stated that multiple salvage chemotherapy regimen was effective in patient group with GTN in whom standard treatment regimens had been unsuccessful and that liposomal doxorubicin was an effective treatment regimen.

Saul et al. (25) showed that the presence of folic acid receptors modified the effects of liposomal doxorubicin at doses of 10 µM in KB, C6 glioma cells and E9 cortex cells. Similarly, Lee and Low (26) identified folate-PLD complexes at doses of 10 µM, 20 µM and 50 µM and 20 µM folic acid and stated that these complexes had higher affinity in targeting cancer cells.

Pariente et al. (27) evaluated the in vitro effect of melatonin on the cytotoxic and pro-apoptotic actions of chemotherapeutic agents including cisplatin (CIS), 5-fluorouracil (5-FU) and DOX in cervical cancer HeLa cells. It was shown that melatonin increases the cytotoxic effect of the chemotherapeutic agents, caspase-3 activation in CIS- and 5-FU-challenged cells and also elevated the ratio of the cells which enter mitochondrial apoptosis due to the production of reactive oxygen species. HeLa cell viability was approximately 73.1% after administration of 20 μM doxorubicin alone whereas this decreased to 57.9% when administered in combination with 1 mM melatonin. Doxorubicin alone was found to cause an approximately 12-fold elevation in caspase-3 activity whereas a 17-fold elevation was shown when administered in combination with 1 mM melatonin. The administration of 20 μM CIS alone induced an approximately 53% apoptosis in HeLa cells whereas an apoptosis of 73% was demonstrated when 20 μM CIS and 1 mM melatonin were concomitantly administered. It was concluded that indoleamine can be applied as a potentially strong synergistic agent in the treatment of cervical cancer (27).

Similarly, it has been shown that in vitro melatonin potentiates cytotoxic and apoptotic effects of CIS and particularly 5-FU by stimulating MT3 receptor in the human colorectal adenocarcinoma cells (HT-29) and HeLa cells (28). Cell viability rates in HT-29 and HeLa cells were approximately 30.7% and 22.7% respectively, after administration of 1 mM 5-FU alone, whereas these were approximately 11.1% and 10.7% after combined administration of 1 mM 5-FU and 1 mM melatonin, respectively. Apoptosis rates in HT-29 and HeLa cultures were approximately 29% and 45% after administration of 20 μM CIS alone, whereas these rates were 45% and 50% after administration in combination with 1 mM melatonin, respectively. Apoptosis rates induced by 5-FU alone were approximately 46% and 47% whereas these rates were 71% and 65% after concomitant treatment of 5-FU and 1 mM melatonin, respectively. These increased rates of apoptosis after administration in combination with melatonin was significant (p<0.05). A further study reported that melatonin increases the efficacy of CIS and 5-FU in HT-29 cells. Apoptosis rate after administration of 1 mM 5-FU alone was 24.1% whereas apoptosis reached 30% when administered in combination with 1 mM melatonin. It was concluded that melatonin increased the sensitization of HT-29 cells to 5-FU treatment and thus indoleamine could be used as a potential chemosensitizing agent in the treatment of adenocarcinoma (29).

There are studies showing that carotenoids are protective against head, neck, mouth, skin, lung and other malignancies and hematopoietic diseases. Furthermore, increased dietary intake of carotenoids (beta-carotene, alfa-carotene, lycopene, beta cryptoxanthin, lutein and zeaxanthin) was associated with lower oesophagus cancer risk. Most of the in vitro studies recently focused on anti-carcinogenic mechanism of beta-carotene in lung, liver and blood cells. In some animal studies, alfa-carotene demonstrated a higher suppressor activity on liver, lung, skin and colon carcinogenesis compared to beta-carotene (30).

Studies have shown that the molecular protective mechanisms of carotenoids in isolated human cell culture include: (1) stopping the cell cycle in G1/G0 phase by decreasing cycline D1 levels; (2) apoptosis induction downregulating survivin levels; (3) increase in cellular gap junction communication and (4) angiogenic effect through modulation of various cytokines including decreased interleukin-6 (IL-6), IL-1b, tumour necrosis factor alpha and granulocyte-macrophage colony-stimulating factor levels and increased IL-2 and TIMP metallopeptidase inhibitor 1 (TIMP-1) levels (30,31). Chemotherapeutic effects via similar mechanisms have been reported for all-trans retinoic acid (ATRA), another vitamin A analog (32). Beta-carotene is a modified antioxidant and reduces oxidative stress (33). Researches have indicated that oxidative stress plays a critical role in the etiopathogenesis of GTD (34,35,36).

Dutta et al. (30) showed that alfa and beta-carotene, in 5 and 10 µM doses respectively, synergistically decreased cell proliferation and DNA synthesis in oesophagus epithelial cell culture (HEE) and squamous cancer cell culture (HESC) when used in combination and identified that early administration could be of benefit in oesophagus cancer treatment, for example in Barret oesophagus, and a lower dose of beta-carotene could be synergistically used with alfa-carotene for protection against oesophagus malignancy.

Wang et al. (37) indicated that minimum beta-carotene concentration for significant cell proliferation of EC9706 cells was 40 µM. However, in our study the minimum beta-carotene concentration for significant decrease in cell proliferation was 1 μg/mL (approximately 1.86 µM). Hurst et al. (38) reported that high doses of beta-carotene caused decreased mitochondrial function through unidentified mechanisms in human K562 erythroleukemic and 28SV4 retinal pigment epithelial cells. They noted that there were insufficient clinical studies evaluating beta-carotene toxic effects on human.

Gloria et al. (39) in a cell culture model of breast cancer identified that use of beta-carotene doses of 0.5, 1, 2.5, 5, and 10 µM had dose-dependent apoptosis and necrosis-enhancing effects in MCF-7, MDA-231 and MDA-235 cell lines. Moreover, Wang et al. (40) showed doxorubicin use at doses starting from as low as 0.5 µM induced apoptosis in PA-1 ovarian teratocarcinoma cells and MCF-7 cells. Osman et al. (41) indicated that doxorubicin at 0.25 µg/mL caused early apoptosis at a rate of 78%, resveratrol 15 µg/mL caused early apoptosis at a rate of 76% and use in combination caused early apoptosis at a rate of 90%. Previously, Sel et al. (42) had reported that ATRA was an effective drug on JAR and JEG-3 cell lines due to decreasing oxidative stress.

To the best of our knowledge this is the first report presenting the effect of beta-carotene in combination with PLD on JAR and JEG-3 CC cells in vitro. When beta-carotene was administered alone and at increasing doses, its apoptotic effects on JAR cell significantly increased. However, its apoptotic effects on JEG-3 cell was not significant. When PLD was administered alone at increasing doses its apoptotic effects on both JAR and JEG-3 cells significantly increased. When increasing concentrations of PLD were combined with a fixed concentration of beta-carotene there was a significant increase in apoptotic effect in both JAR and JEG-3 cells.

## Conclusion

This is the first report, as far as we are aware, presenting the effects of beta-carotene and PLD in combination on JAR and JEG-3 human CC cell line models. The effects were assessed by measuring the concentrations of β-hCG and H-hCG. Apoptotic data showed that beta-carotene and PLD combination has a synergistic effect and may be a viable option for treatment of multiple drug resistant human CC. The results of this study suggest that vitamin A supplementation may have a preventive role in human CC in the future. However, these were in vitro studies and the results should be confirmed in animal trials and, if justified, clinical studies, as the effects of the drugs and the effects in combination may differ in in vivo systems.

## Figures and Tables

**Table 1:**
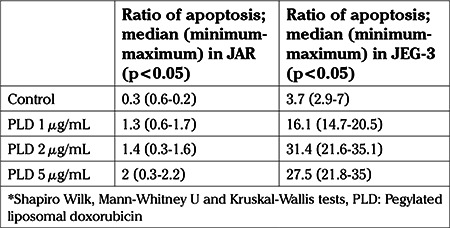
Ratio of apoptosis (%) on control group and after application of PLD to JAR and JEG-3 cell lines

**Table 2:**
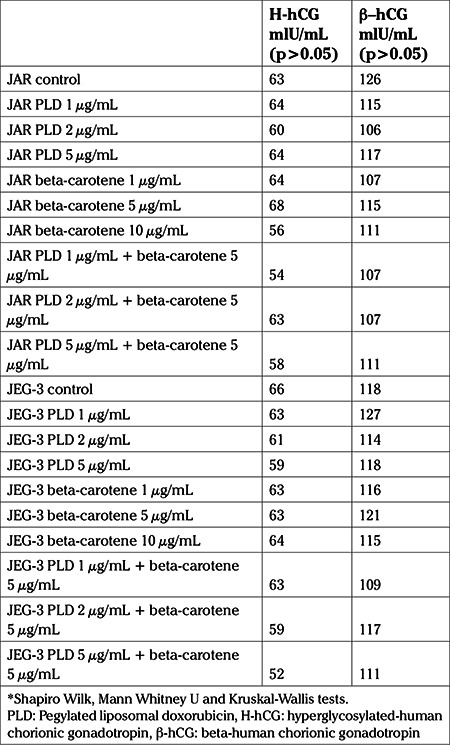
H-hCG and β-hCG levels on control group and after application of pegylated liposomal doxorubicin, beta-carotene and combined doses to JAR and JEG-3 cell lines

**Table 3:**
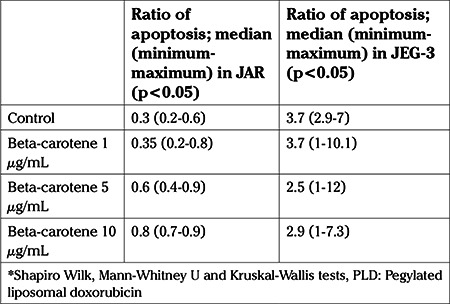
Ratio of apoptosis (%) on control group and after application of beta-carotene to JAR and JEG-3 cell lines

**Table 4:**
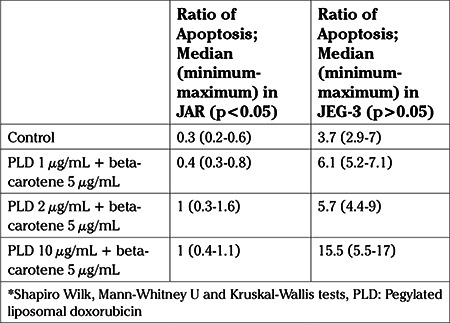
Ratio of apoptosis (%) on control group and after application of combined doses of pegylated liposomal doxorubicin and beta-carotene to JAR and JEG-3 cell lines

**Graphic 1:**
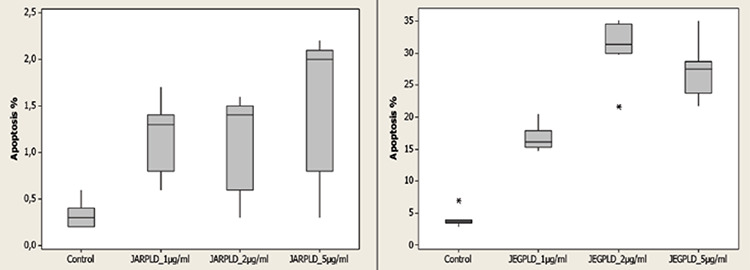
Ratio of apoptosis (%) on control group and after application of pegylated liposomal doxorubicin to JAR and JEG-3 cell lines (p<0.05) *Shapiro-Wilk, Mann-Whitney U and Kruskal-Wallis tests, PLD: Pegylated liposomal doxorubicin

**Graphic 2:**
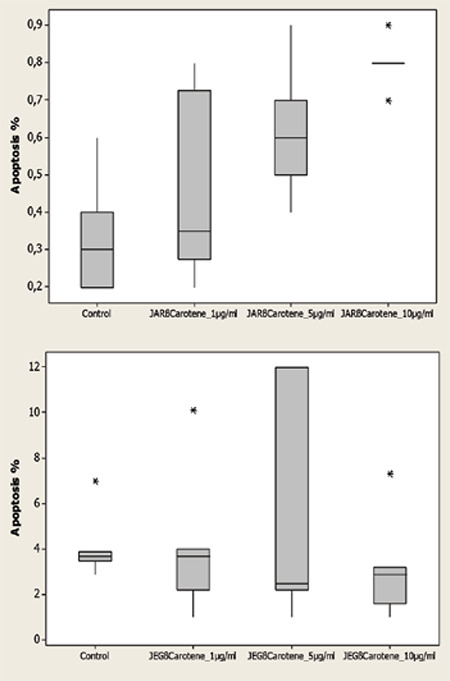
Ratio of apoptosis (%) on control group and after application of beta-carotene to JAR and JEG-3 cell lines (p<0.05, p>0.05, respectively) *Shapiro-Wilk, Mann-Whitney U and Kruskal-Wallis tests

**Graphic 3:**
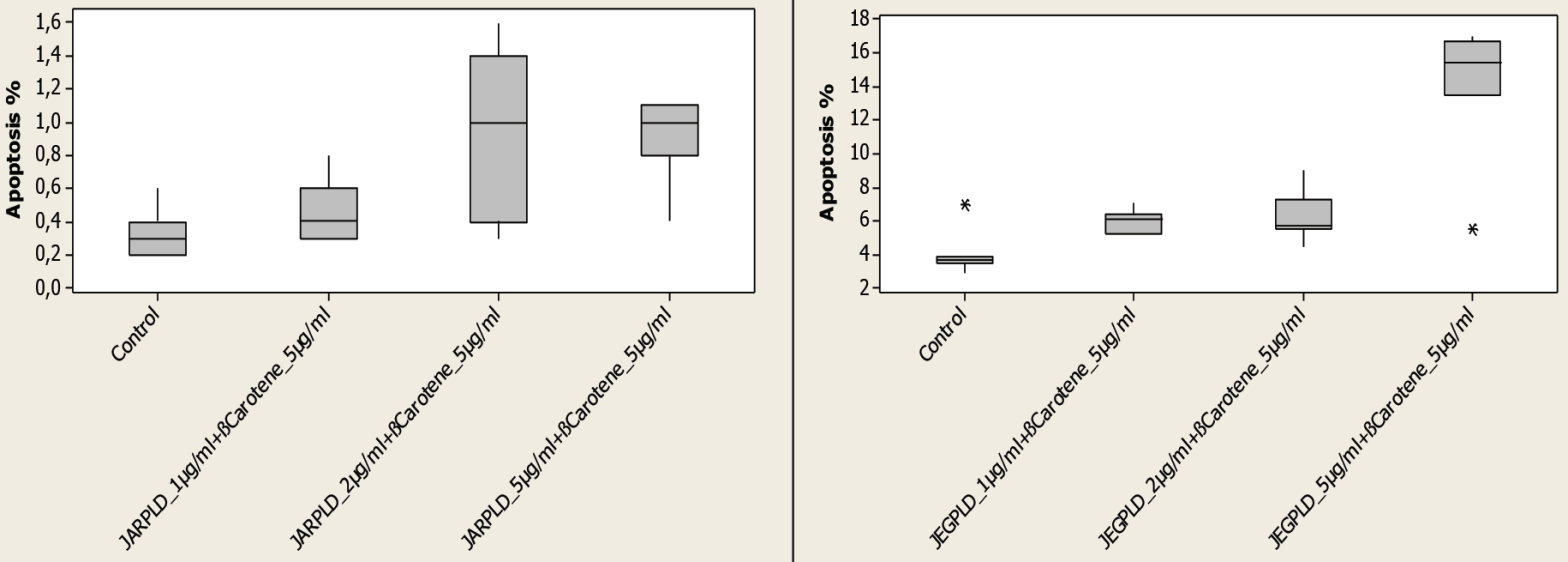
Ratio of apoptosis (%) on control group and after application of combined doses of pegylated liposomal doxorubicin and beta-carotene to JAR and JEG-3 cell lines (p<0.05) *Shapiro-Wilk, Mann-Whitney U and Kruskal-Wallis tests, PLD: Pegylated liposomal doxorubicin
